# The Fruits of "The Hospital's" Teaching

**Published:** 1917-06-16

**Authors:** Frederic G. D'Aeth

**Affiliations:** Liverpool Council of Voluntary Aid, 2 Exchange Street East, Liverpool.


*To the Editor of* THE HOSPITAL.

### The Fruits of "The Hospital's" Teaching.

*To the Editor of* THE HOSPITAL.

SIR,—My attention has been drawn to your references to our work under the above heading in your current issue. I am much interested to see the latest addition to the list of sires who desire to claim parentage over this lusty infant! Some day I will have a list of them drawn up and send it round to the parents with the suggestion that they explain their obviously immoral conduct! Fortunately, the child itself possesses its own pedigree, so far as the case of an institution is possible!

However, quite apart from this raillery, I should like to thank you for the appreciation of our work. I may say that we have filed THE HOSPITAL for the past five years, and have found it very valuable for all matters relating to the medical world.—Yours very faithfully,

FREDERIC G. D'AETH.

Liverpool Council of Voluntary Aid,
2 Exchange Street East, Liverpool.
June 8, 1917.

